# Humoral response in Leishmaniasis

**DOI:** 10.3389/fcimb.2022.1063291

**Published:** 2022-12-12

**Authors:** Luciana Conde, Gabriela Maciel, Gustavo Meira de Assis, Leonardo Freire-de-Lima, Dirlei Nico, André Vale, Célio Geraldo Freire-de-Lima, Alexandre Morrot

**Affiliations:** ^1^ Instituto de Biofísica Carlos Chagas Filho, Universidade Federal do Rio de Janeiro, Rio de Janeiro, Brazil; ^2^ Instituto de Microbiologia Paulo de Góes, Universidade Federal do Rio de Janeiro, Rio de Janeiro, Brazil; ^3^ Faculdade de Medicina, Universidade Federal do Rio de Janeiro, Rio de Janeiro, Brazil; ^4^ Instituto Oswaldo Cruz, FIOCRUZ, Rio de Janeiro, Brazil

**Keywords:** *Leishmania*, humoral response, leishmaniasis, complement system, B lymphocytes

## Abstract

Leishmaniasis presents different types of clinical manifestations that can be divided into cutaneous leishmaniasis and visceral leishmaniasis. The host’s immune system, associated with genetic and nutritional factors, is strongly involved in the evolution of the disease or parasite escape. Humoral immunity is characterized by the production of antibodies capable of promoting neutralization, opsonization, and activation of the complement system. In this scenario, B lymphocytes produce antibodies that play an important role in Leishmania infection although neglected for a long time. Thus, relevant aspects in the establishment of Leishmania infection will be addressed, highlighting the importance of humoral immunity during the entire process of Leishmania infection.

## Introduction

Leishmaniasis is a complex of diseases caused by flagellated protozoa of the genus *Leishmania* (order Kinetoplastida, family Trypanosomatidae) with different clinical manifestations (Kaufer et al., 2017). The protozoan is inoculated into the vertebrate host during the blood meal of infected female sandflies and can generate the disease symptomatically or asymptomatically (WHO, 2022). Leishmaniasis has two main forms with different clinical manifestations, cutaneous leishmaniasis (CL) and visceral leishmaniasis (VL). The symptomatology is determined by a combination of factors, relating to the host, the parasite, and the vector, mainly the *Leishmania* species and the vertebrate host’s immune response to infection (Kaufer et al., 2017).

CL is the most common form of leishmaniasis. It can occur in three different types: localized cutaneous leishmaniasis (LCL); diffuse cutaneous leishmaniasis (DCL); and mucocutaneous leishmaniasis (ML) (Reithinger et al., 2007). Collectively these three cutaneous forms can be called cutaneous leishmaniasis (CT). LCL is the mildest form of leishmaniasis, its clinical manifestation is characterized by one or multiple lesions that can ulcerate in exposed parts of the body, usually rounded and with raised edges (Gontijo and de Carvalho, 2003). DCL is the least common form (Sampaio et al., 2021), characterized by the development of multiple nodules without ulceration that can affect the entire body. ML is a form restricted to Latin America, after skin lesions, the disease spreads to the mucous membrane of the nose, mouth, and throat, where subsequently there is the formation of ulcers in the mucosa that destroy the nasal septum, lips, and nose. palate, leading to deformations that disfigure the face of the infected individual (Steverding, 2017). The main species of *Leishmania* involved in the localized cutaneous manifestation are: *Leishmania braziliensis*, *Leishmania amazonensis*, *Leishmania panamensis*, *Leishmania lainsoni*, *Leishmania guyanensis*, *Leishmania tropica*, *Leishmania major*, *Leishmania mexicana*; in diffuse cutaneous manifestation: *Leishmania amazonensis*; in the mucocutaneous manifestation: *Leishmania braziliensis* (Desjeux, 2004; Reithinger et al., 2007).

Meanwhile, VL is the most severe form of the disease (Chapman et al., 2015). More than 90% of VL cases occur in the African Continent, in the Indian Subcontinent and in Latin America (Chappuis et al., 2007). Whereas in North Africa and in Latin America VL is commonly attributed to *Leishmania infantum*, cases in East Africa and in the Indian Subcontinent are usually linked to Lesihmania donovani (Lukes et al., 2007). VL is also known as Kala-azar, an Indian name for “black fever”, due to the prolonged febrile manifestation and hyperpigmentation associated with the disease. It is characterized by the infection of phagocytes and of the reticuloendotelial system, leading to the infection of many anatomically associated sites, such as lymph nodes, spleen and liver (WHO, 2022).

Although most research groups focus on the study of cellular responses to Leishmaniasis for reasons that will be discussed subsequently, the challenges in control, treatment and vaccine formulation highlight the necessity of better understanding and discussing aspects of humoral immunity.

## General aspects and immune system cells involved in the initiation of *Leishmania* infection

The immune response against the infection is dependent on several factors, such as its antigenicity, the host’s immune system, and the parasite load (Santos-Gomes et al., 2002). After inoculation of the parasite by the vector into the vertebrate host, *Leishmania* benefits from a pro-inflammatory environment induced by the vector’s saliva for its intracellular infection, which, through chemoattraction, attracts phagocytic cells to the site of infection (Chagas et al., 2014). The first cells to arrive at the site of infection and actively phagocytose the promastigote forms of *Leishmania* are neutrophils (Muller et al., 2001; Mollinedo et al., 2010). However, the most important cells for parasite replication and the establishment of infection are macrophages. Macrophages confine phagocytosed *Leishmania* in a phagolysosome, a low pH organelle filled with lytic enzymes (Podinovskaia and Descoteaux, 2015). The main leishmanicidal mechanisms of the macrophage are the production of reactive oxygen species (ROS) and reactive nitrogen species (RNS), these processes are extremely important for the elimination of the parasite without damage to the host cell (Iles and Forman, 2002; Fang, 2004). The inhibition of these mechanisms is the main evasion strategy of the parasite. The *Leishmania* metalloproteinase gp63 inhibits oxidative stress by interfering with the induced nitric oxide synthase (iNOS) and NADPH oxidase 2 (NOX2) signaling pathways of macrophages (Olivier et al., 2012).

Although innate immunity is associated with the elimination of the intracellular parasite, recently important aspects regarding humoral immunity have been raised during *Leishmania* infection.

## Humoral response in leishmaniasis

Humoral immunity is mediated by antibodies secreted by B cells (Mauri and Bosma, 2012). During the immune response, antibodies are capable of neutralization, opsonization, and activation of the complement system (CS). In leishmaniasis, the importance of CS activation is commonly highlighted since it is the first barrier faced by *Leishmania* in the vertebrate host. The parasite can evade the CS, preventing its lysis through surface molecules such as LPG and gp63 (Gurung and Kanneganti, 2015).

For a long time, B lymphocytes were neglected in *Leishmania* infection, as these parasites are obligatorily intracellular (Bates and Rogers, 2004). However, studies have already demonstrated the exacerbation of the B lymphocyte’s response to infection by some *Leishmania* species favoring the parasite (Firmino-Cruz et al., 2019). Studies with B-cell-deficient mice have shown that symptoms appear later and with less severe lesions than in control mice (Smelt et al., 2000; Wanasen et al., 2008).

Deak and colleagues demonstrated that polyclonal activation of B cells in the course of infection leads to disease exacerbation. Through the use of B-cell-deficient mice and adoptive transfer of specific or non-specific IgM and IgG, a return to the susceptibility phenotype was observed in JnD Balb/c resistant mice (Deak et al., 2010). The correlation between B cells and a poor prognosis in leishmaniasis was also evidenced by the work of Omachi et al., demonstrating that animals deficient in B cell activating factor (BAFF) can suppress the splenomegaly characteristic of the disease in the experimental model of VL with *L. donovani*, but not hepatomegaly (Omachi et al., 2017). In a previous study, the same group demonstrated an increase in serum levels of BAFF in patients with visceral leishmaniasis, where the mean value of BAFF in Brazilian patients was 4.3 higher than the mean of controls (Goto et al., 2014). The magnitude of this increase in serum BAFF levels is equivalent to the increase demonstrated in patients with systemic sclerosis (Matsushita et al., 2006) and Sjorgen syndrome (Groom et al., 2002).

## Prevalence of different immunoglobulin classes may point to different stages of the disease and different clinical outcomes

One of the most discussed immunological aspects of both cutaneous (Castellano et al., 2009) and visceral (Heinzel et al., 1989; Wang et al., 1994) leishmaniasis is the dynamics between Th1 and Th2 responses, in which a dominant Th2 response, stimulated by the preponderant presence of cytokines such as interleukin-10 (IL-10) and interleukin-4 (IL-4), would suppress the effector profile of a Th1 response and clamp down the classical activation of macrophages (M1). This would favor the parasite with the dominance of anti-inflammatory/pro-resolutive M2 macrophages, which not only block more aggressive responses that could help parasite clearance but are also susceptible cells in which the entry of *Leishmania* promastigotes is facilitated (Heinzel et al., 1993; Farrow et al., 2011; Lee et al., 2018).

In view of a such well established paradigm and the unfavorable effects of B cell responses to the host that will be discussed below, humoral responses in Leishmaniasis have not been a big focus of interest in this field of research. However, B cells can function not only as antibody-secreting cells, but they can also modulate the immune response through antibody-independent mechanisms, such as antigen presentation and secretion of cytokines and chemokines (Myers, 1991; Lund, 2008).

The presence of B cells and their polyclonal activation has been directly correlated with a poor prognosis of the disease, recent studies have directed efforts to demonstrate the role of regulatory B cells (Breg) in the course of the disease (Soares et al., 2017). Recently, it was demonstrated that the incubation of B cells with amastigote forms of *L. infantum* is capable of activating subpopulations of human B cells with an immunoregulatory phenotype that secretes IL-10 in a dose-dependent manner, inhibiting the activation and proliferation of CD4+ T cells (Andreani et al., 2015). Type 1 B cells (B-1) have also been implicated in susceptibility in experimental visceral leishmaniasis infection with increased IL-10 and it has been shown that Balb/XID mice (B-1 cell deficient) have lower serum IL-10 and less parasite load in the spleen compared to the control. The transfer of B-1 cells to IL-10 knockout animals led to increased susceptibility to *L. chagasi* infection (Gonzaga et al., 2015). In other infections with protozoa, such as *Trypanosoma cruzi*, studies have shown that antibodies are responsible for the survival of susceptible animals in the early stages of the disease and the maintenance of low levels of parasitemia in the chronic phase (Umekita et al., 1988; Bermejo et al., 2011). Thus, parasite-specific immune response is insufficient to eradicate the disease, allowing infection in the chronic phase.

The dominant cytokine profile also impacts antibody production both in quality and quantity, as it can direct B cells to engage in class switch recombination of the immunoglobulin gene (Snapper and Paul, 1987). Although high titers of antileishmanial antibodies are characteristically present in the visceral forms of the disease (Behforouz et al., 1976; Carvalho et al., 1985; Sacks and da Silva, 1987), a feature accompanied by diminished cellular response (Castes et al., 1983; Cillari et al., 1988), this antibody abundance is usually not capable of promoting protection of the host (Nylen and Gautam, 2010). In fact, B cell activity is often described as detrimental in the context of leishmaniasis: IgG-coated *L. major* amastigotes could be internalized more efficiently by murine macrophages, subsequently inducing IL-10 production (Kane and Mosser, 2001) which has been described as detrimental for, among other effects, supposedly aiding the shift from a predominantly Th1 profile to a predominantly Th2 (Ghalib et al., 1995; Revaz-Breton et al., 2010). Additionally, Fc deficient mice infected by *L. amazonensis* were observed to produce less IL-10 and to be less susceptible to infection (Buxbaum and Scott, 2005). High antibody titers have also been reported in association with disease severity in mice experimentally infected with *L. amazonensis* (Wanasen et al., 2008).

On the other hand, the adoptive transfer of IL-10-producing B-1 cells to infected mice did not impact disease outcomes (Firmino-Cruz et al., 2020). It has been shown that T cells themselves can be a source of IL-10 during visceral leishmaniasis in an antigen-dependent manner, determining infection aftermath in mice (Schwarz et al., 2013). This, of course, impacts vaccine development, as it would be necessary to induce a response that would exclude the activation of IL-10-producing T cells while still promoting the adaptive cellular response.

Still, more attention has been given to the potentially detrimental contribution of B cells in the context of leishmaniasis. While high anti-*Leishmania* IgG titers have been correlated to mucosal leishmaniasis severity (de Lima et al., 2021), a correlation between high levels of *Leishmania*-specific IgA and IgE seem to have contributed to more severe forms of American tegumentary leishmaniasis in the context of *L. panamensis* infection (O’Neil et al., 1993). Likewise, abundant IgG and IgM, forming immune complexes with complement factors of the classical and terminal pathways, have been implicated in Leishmaniasis-Associated Membranoproliferative Glomerulonephritis (Sethi et al., 2016). Furthermore, a case of *L. infantum* reactivation with secondary IgA nephropathy has recently been described (Grewe et al., 2022).

Nevertheless, understanding the dynamics of antibody production in leishmaniasis may be an important prognostic tool. Steady levels of IgM, IgE, and IgG4 following drug therapy can be suggestive of disease persistence and potential clinical relapse (Anam et al., 1999). This is especially important considering the rise of strains resistant to pentavalent antimonials (Thakur et al., 1997; Rugani et al., 2019; Andrade et al., 2020).

Conversely, the importance of B cells and humoral response in protective responses to *Leishmania* should not be completely discarded. Studies characterizing B cell clones (through the sequencing of the rearranged, and potentially somatically hypermutated, immunoglobulin gene segments) and the subset to which these clones belong are still needed. For instance: while IgG1 against *L. infantum* was correlated to asymptomatic infection and IgG2 to disease manifestation by one group (Reis et al., 2006), another group observed that even though asymptomatic dogs infected with *L. infantum* had lower levels of anti-*Leishmania* IgG2, dogs protected against the disease through vaccination with Leishimmune^®^ (Fort Dodge Animal Health) had high levels of anti-*Leishmania* IgG2 (Oliveira et al., 2009). Interestingly, although high titers of IgE have been implicated in disease activity in the context of VL, high titers of IgE in CL have been observed to be correlated with a diminished number of skin ulcers, although positively correlated with bigger Montenegro’s reaction size (Atta et al., 2002). These data suggest that protective action of IgG2 and IgE is context dependent and that maybe the binding site characteristics of the antibodies is more decisive than immunoglobulin class.

## Complement system

The complement system plays an important role in innate immune defense, consisting of about 35 proteins that may be present in the plasma or on the plasmatic membrane surface of some cell types (Trouw and Daha, 2011; Ambrosio et al., 2021). Previous studies discuss that several proteins that constitute the complement system are synthesized in the liver, about 7 proteins can be synthesized by human skin fibroblasts (Katz et al., 1989) and it is currently known that dendritic cells are capable of synthesizing C1q, C3, Factor I, Factor B and complement receptors 3 and 4 (Reis et al., 2007). Rcent studies shed light on the contribution of adipose tissue to the activation of the complement system through the production of complement factor D. Factor D is a serine protease that will play a fundamental role in generating the C3 convertase, after cleaving factor B, activating the alternative complement pathway (Sekine et al., 2022).

In addition to its role as an effector mechanism of the innate immune system, complement also plays an important role in the formation of the adaptive immune response. This occurs because these proteins can interact with each other, triggering a proteolytic cascade or with other molecules, such as antibodies. Activation of the complement system can occur through three distinct pathways: the classical pathway (Cooper, 1985), the lectin pathway (Sato et al., 1994), and the alternative pathway (Soothill and Harvey, 1977). All these pathways converge to a common point resulting in the activation of the C3 component and its deposition on the surface of a pathogen (Walport, 2001). Regardless of the pathway of activation of the complement system, all 3 lead to the formation of the C3-convertase complex that will then initiate the proteolytic cascade favoring the formation of the Membrane Attack Complex (MAC) that causes osmotic lysis of the pathogen (Trouw and Daha, 2011).

Protozoa of the genus *Leishmania* are obligate intracellular parasites that need to be phagocytosed and survive within phagocytic cells of mammals, such as neutrophils and macrophages (Podinovskaia and Descoteaux, 2015). To survive the hostile environments faced throughout its life cycle, *Leishmania spp* expresses unique molecules such as glycoinositolphospholipids (GIPLs), which are glycoconjugates known to be the main component of the parasite’s surface, lipophosphoglycan (LPG) and metalloprotease GP63 (Davies et al., 1990). More recent studies with *L. infantum* investigated how the LPG molecule influences the initial establishment of infection during interaction with human neutrophils in an *in vitro* experimental environment. They observed that mutant parasites that did not express LPG had a reduced viability and that this was related to an increased lysosomal fusion in the neutrophils evaluated by confocal microscopy (Quintela-Carvalho et al., 2022). Other remarkable adaptive mechanisms include inhibition of phagosome-endosome fusion (Desjardins and Descoteaux, 1997), expression of hydrolytic enzymes, modulation of cell signaling pathways (Eilam et al., 1985), nitric oxide production (Wei et al., 1995), and cytokine induction (Barral-Netto et al., 1992).

While in the bloodstream, the escape of the complement system is an important step in the establishment of the infection, and a mechanism developed by this parasite, both in metacyclic promastigotes and in amastigotes, involves the inactivation of C3b, converting it to its inactive form iC3b by the action of the membrane protease GP63, which is the subject of clinical studies for a therapeutic approach (Brittingham and Mosser, 1996; Mosser and Brittingham, 1997). In addition, inactive C3b continues to play the role of opsonization and its deposit on the surface of the parasite increases the chance of phagocytosis, since macrophages and related cells have CR1, CR3, and CR4 receptors that recognize the intact C3b component but also its inactive form (Mosser and Edelson, 1987; Tausk and Gigli, 1990; Brittingham and Mosser, 1996; Mosser and Brittingham, 1997; Lukacsi et al., 2017).

Thus, recent studies have evaluated other mechanisms that the parasite could play to achieve immune escape and maintain opsonization through the inactivation of C3b in iC3b by a pathway other than GP63. It was seen that the parasite can recruit factor H (complement system regulatory protein), which in turn recruits factor I, which acts by cleaving the C3 deposited on the surface of the parasite, promoting its inactivation in iC3b without compromising opsonization and subsequent phagocytosis of the protozoan (Filho et al., 2021).

Another immune escape mechanism was demonstrated *via* the lectin pathway, where mannose-binding lectin (MBL), collectin-11 (CL-11), and ficolins-1 and -3 were shown to bind to the surface of *L. infantum* promastigotes (both LPG and GIPL) when exposed to 20% NHS (normal human serum). These molecules can recognize pathogen-associated molecule patterns (PAMPs) on the surface of *L. infantum* promastigotes triggering the activation of the lectin pathway, suggesting a role in promoting the host/parasite interaction, leading to important events such as phagocytosis and macrophage activation in the initial infection (Ambrosio et al., 2021). Furthermore, it has been shown that high levels of serum mannose-binding lectin (MBL) are associated with modulation in macrophage function, increasing the susceptibility to Leishmaniasis infection (Santos et al., 2001). It has been shown that *Leishmania donovani* is able to inhibit the lectin pathway through the activity of its Inhibitor of Serine Proteases 2 (LdISP2), preventing the formation of the MAC by reducing the formation of C3 and C5 convertases (Verma et al., 2018). Finally, studies have shown that genetic modifications in complement genes, such as single nucleotide polymorphisms (SNPs), can influence host susceptibility to these parasites (Tirado et al., 2021).

## Conclusion

Divergent observations, such as that IgG2 can be protective or detrimental in the context of *L. infantum* infecting dogs, may point to the possibility that the complementarity-determining regions of the antibodies are more relevant than the immunoglobulin class itself in determining disease outcome. Although some of the studies show that mice lacking B cells are resistant to several forms of leishmaniasis, B cell depletion brings a huge variety of physiological imbalances, which would not be desirable in therapeutic applications to humans and animal companions. In the light of the fact that any vaccine that acts on T cells will affect B cell activity through cognate interactions, screening of the protective B cell repertoire and subpopulation distribution in the context of leishmaniasis is of the utmost importance.

The unique adaptive mechanisms developed by *Leishmania spp* to evade immune responses includes the ability to inhibit the complement system of mammalian hosts. It is not clear, however, if antibodies targeting surface molecules and enzymes involved in this process could impair such evasion. In light of the recent discovery that *Leishmania donovani*’s LdISP2 is able to inhibit C3 and C5 convertase formation, an interesting question would be if antibodies that bind to LdISP2 would be protective or attenuate the disease *in vivo*. In case such antibodies are protective, another interesting question would be if Immunoglobulin class is determinant in such protection. Understanding if specific B cell subpopulations are more implicated in the secretion of antibodies would also be compelling, as this may determine what would be the best adjuvants for an immunization protocol. These types of data may be useful in future vaccine designs that take not only cellular, but also humoral adaptative responses into consideration.

## Author contributions

LC, GaM, GuM, LF-D-L, DN, AV, CF-D-L, AM wrote the manuscript equally. All authors contributed to the article and approved the submitted version.
